# 5’UTR sequences influence protein levels in *Escherichia coli* by regulating translation initiation and mRNA stability

**DOI:** 10.3389/fmicb.2022.1088941

**Published:** 2022-12-21

**Authors:** Fan Chen, Muriel Cocaign-Bousquet, Laurence Girbal, Sébastien Nouaille

**Affiliations:** TBI, CNRS, INRAE, INSA, Université de Toulouse, Toulouse, France

**Keywords:** mRNA stability, translation initiation, post-transcriptional regulation, gene expression regulation, protein level regulation, *Escherichia coli*

## Abstract

A set of 41 synthetic 5’UTRs with different theoretical translation initiation rates were generated to explore the role of 5’UTRs in the regulation of protein levels in *Escherichia coli*. The roles of the synthetic 5’UTRs in regulating the expression of different reporter genes were analyzed *in vivo*. Protein levels varied substantially between the different constructs but for most of the 5’UTRs, protein levels were not correlated with theoretical translation initiation rates. Large variations in mRNA concentrations were measured with the different 5’UTRs even though the same concentration of transcription inducer was used in each case. 5’UTRs were also found to strongly affect mRNA stability, and these changes in mRNA stability often contributed to observed differences in mRNA concentration. Unexpectedly, the effect of the 5’UTRs on mRNA half-lives was found to vary depending on the downstream reporter gene. These results clearly demonstrate that 5’UTRs contribute to gene expression regulation at the level of translation initiation and of mRNA stability, to an extent that depends on the nature of the downstream gene.

## Introduction

*Escherichia coli* cells adapt their metabolism to changing environments by modifying the expression pattern of their gene repertoire. Gene expression control encompasses a wide range of transcriptional and post-transcriptional mechanisms that affect different stages of gene expression.

The 5’UTR of mRNA, the transcribed but untranslated region from the transcription start site to the first nucleotide of the translational start codon, is a potential regulatory element for gene expression. 5’UTRs are crucial for efficient translation. Their most important features are generally considered to be the ribosome binding site, the Shine-Dalgarno (SD) sequence, complementary to the anti-SD sequence at the 3’end of 16S rRNA (Shine and Dalgarno, 1974), and A/U richness, which limits the formation of secondary structural elements in the ribosome binding site (Komarova et al., 2002; Kozak, 2005). Strong SD/anti-SD pairing and weak secondary structure in 5’UTRs are the hallmarks of highly expressed genes in *E*. *coli* (Prabhakaran et al., 2015). The regions upstream of the SD sequence and the distance between the SD sequence and the initiation codon also play important roles (Hartz et al., 1991; Komarova et al., 2002; Osterman et al., 2013). Translation initiation of particular mRNAs is also affected by 5’UTR binding of regulatory intracellular molecules, metabolites in the case of riboswitches (Tucker and Breaker, 2005), small RNA (sRNA) molecules (Gottesman and Storz, 2011; Lalaouna et al., 2013; Wagner and Romby, 2015) and RNA-binding proteins (CsrA, Hfq, and ProQ; Folichon et al., 2003; Holmqvist et al., 2018; Holmqvist and Vogel, 2018; Romeo and Babitzke, 2018).

The effect of 5’UTRs on translation indirectly influences mRNA stability (Iost and Dreyfus, 1995; Komarova et al., 2005) because limiting translation initiation destabilizes mRNA. Mutating the ribosome binding site of the *lacZ* reporter gene has been shown to reduce the efficiency of ribosome binding, leading to a decrease in *lacZ* mRNA stability (Yarchuk et al., 1992; Iost and Dreyfus, 1995). The structured motifs of 5’UTRs also modulate the efficiency of translation initiation by sequestering/exposing the SD sequence, thereby reducing/increasing its accessibility to ribosomes (Chowdhury et al., 2006). The protective effect of high ribosomal occupancy is thought to arise from ribosomes outcompeting RNase E, thereby preventing 5′-end-dependent degradation of the transcript (Dreyfus, 2009). mRNA stability is also directly regulated by the binding of sRNAs and RNA binding proteins to 5’UTRs, which can activate or prevent RNase E scanning and cleavage (Ikeda et al., 2011; Prevost et al., 2011; Vakulskas et al., 2015; Richards and Belasco, 2019).

In this study, to further investigate the role of 5’UTR sequences in gene expression regulation in *E*. *coli*, we measured the effects of a large set of synthetic 5’UTR sequences on mRNA and protein levels and mRNA stability. The synthetic 5’UTR sequences were chosen to cover a large range of theoretical translation initiation rates and were placed under the control of an inducible promoter and fused to different reporter genes. This approach has several advantages. (i) The use of synthetic 5’UTRs instead of native sequences limits the risk of interference with the binding of intracellular regulatory molecules. (ii) Length effects are avoided because the considered 5’UTRs are identical in length. (iii) No knowledge of specific regulatory elements within the 5’UTR sequence is required because the entire 5’UTR sequence is replaced in each case. (iv) Similar levels of transcription initiation can be applied to reduce differences in mRNA synthesis between the constructs. And (v), the interplay between the effects of the 5’UTRs and of the downstream gene can be explored. Results show that for most of the synthetic 5’UTRs, the theoretical rate of translation initiation is not a good predictor of protein levels. Many 5’UTRs were found to regulate mRNA levels by altering the stability of the transcript in a downstream gene dependent manner. Overall, our results demonstrate that 5’UTRs act as “regulatory-hubs” of gene expression in close interaction with the downstream gene.

## Materials and methods

### Design of synthetic 5’UTR sequences

RBS Calculator was used to design synthetic 5’UTR sequences with different translation initiation rates (Salis, 2011). RBS Calculator uses a statistical thermodynamic model considering Gibbs free energies for key molecular interactions in translation initiation to give an estimation of translation initiation rate. RBS Calculator was used to generate synthetic 5’UTR sequences (containing a RBS) with rationally controlled translation initiation rates over a 100,000-fold range. The 5’UTRs were designed based on the first 150 nucleotides of the *lacZ* coding sequence. The reference 5’UTR was taken from the P_BAD_-*lacZ* control plasmid from the Invitrogen P_BAD_-his/myc expression system used for the production of heterologous proteins in *E*. *coli*. This 5’UTR is 33 nt long with an efficient SD sequence (GGAGG) and an RBS index of 33,000 arbitrary units (AU). Ten RBS indexes were targeted to cover a wide range of values (100, 250, 500, 1,000, 1,500, 2,500, 5,000, 33,000, 66,000, and 100,000 AU). For each RBS index, 200 unique 33 nt 5’UTR sequences were generated by RBS Calculator and from 3 to 6 sequences were manually selected from each class, with high GC% and with low GC%. One additional criterion was that the sequences should not have a strong folding propensity (minimum free energy calculated using the mfold software) within the 5’UTR sequence or at the beginning of the *lacZ* coding sequence. Including the reference sequence, this procedure yielded a set of 41 synthetic 5’UTR sequences covering a wide range of RBS indexes (Supplementary Table S1).

### Plasmid constructions

The synthetic 5’UTRs were introduced in place of the reference 5’UTR in the P_BAD_-*lacZ* plasmid by PCR. For each 5’UTR, a primer was designed to hybridize with the recipient vector at the ATG of the *lacZ* CDS at the 3′ end and was extended with the 5’UTR specifying sequence at the 5’end. These were used in combination with a reverse primer hybridizing to the promoter region from the nucleotide upstream of the +1 transcription start site. The full plasmid was amplified by PCR using *Phusion* polymerase (New England Biolabs). The amplicons were gel-purified and the 5’ends were phosphorylated with T4 polynucleotide kinase (New England Biolabs) and self-ligated with T4 DNA ligase. The ligation mix was used to transform *E*. *coli* DH5α cells (New England Biolabs). The primers used for 5’UTR cloning are listed in Supplementary Table S2. Plasmid DNA was isolated using the QIAprep Miniprep kit (Qiagen) and constructs were verified by sequencing (Eurofins). To replace the reporter gene, the CDS from *msfGFP* (encoding monomeric superfolder green fluorescent protein) and *txAbF* (encoding an α-L-arabinofuranosidase from *Thermobacillus xylanilyticus*) were amplified by PCR. The whole plasmid backbones containing the eight selected 5’UTRs fused to *lacZ* were PCR amplified from the end of the *lacZ* coding sequence to the 5’UTR. The amplicons were gel purified and the *msfGFP* or *txAbF* CDS inserts were phosphorylated, ligated with the backbones, and transferred to *E*. *coli* DH5α cells. The fusions between the 5’UTRs and the reporter genes were verified by sequencing.

### Bacterial strains, growth, and induction conditions

All cloning steps were performed using *E*. *coli* DH5α cells (New England Biolabs). Cell cultures were grown in LB or LB-agar. All constructs were transferred into the reference strain (MET 345) for characterization. MET 345 is a derivative strain of DLT 2202 (MG1655 *ΔaraFGH*, *Ωpcp18::araE533*) lacking the chromosomal copy of *lacZ* (Ah-Seng et al., 2013; Nouaille et al., 2017). In this strain, the P_BAD_ promoter is proportionally induced by the concentration of arabinose uniformly in all cells. All the strains were routinely grown in M9 minimal medium (Esquerre et al., 2014) supplemented with ampicillin 100 μg/ml at 37°C, 150 rpm unless otherwise stated. Cultures were inoculated from overnight precultures at an initial OD_600nm_ of 0.1. Induction levels were evaluated by adding arabinose to exponentially growing cultures (OD_600_ = 0.6) with serial dilutions for final concentrations of 0.00001 to 0.1% arabinose. For physiological characterizations, strains carrying *lacZ* and *txAbF* were induced with 0.001% arabinose, and strains carrying *msfGFP* were induced with 0.01% arabinose. Samples were taken 30 min after arabinose induction.

### Measurements of β-galactosidase and α-L-arabinofuranosidase activity

Harvested cells (3 mg dry weight) were washed twice with cold 0.2% KCl, resuspended in 1 ml of breaking buffer (15 mM Tris 400 mM/ tricarballylate 1, 4.5% Glycerol, 0.9 mM MgCl2, 0.2 mM DTT; pH = 7.2) and transferred into screw capped tubes containing 0.1 g of glass beads. The cells were disrupted with a FastPrep-24 instrument (MP Biomedicals) in six cycles (6.5 m/s, 30 s) with 1 min on ice between each cycle. After centrifugation, measurements were performed on the supernatants containing soluble proteins. All measurements were performed in triplicate on three biological replicates. β-Galactosidase activity was determined colorimetrically using o-nitrophenyl-β-D-galactopyranoside (ONPG) as a substrate (Miller, 1972). α-L-arabinofuranosidase activity was measured using discontinuous assays, a colorimetric method (Bissaro et al., 2014) based on 4-Nitrophenyl-α-L-arabinofuranoside (pNP-Araf, colorless), which is hydrolyzed by α-L-arabinofuranosidase and releases p-nitrophenyl (pNP, yellow). Samples (100 μL) were diluted in sodium phosphate (100 mM) and placed in 96-well microplates. Assays were initiated by adding 100 μL of 2X assay buffer. The assay buffer (1×) consisted of 100 mM sodium phosphate at pH 7, 1 mM MgCl_2_, 50 mM β-mercaptoethanol and 10 mM pNP-Araf. Absorbance was measured at 401 nm using a microplate reader (SpectraMax Plus 384). Kinetic readings began immediately after the assay was initiated by adding 2× assay buffer, with absorbance measurements every 20 s for 30 min at 45°C. α-L-arabinofuranosidase activity was measured in units defined by the catalysis of 1 μmol of pNP per minute. Total protein was assayed using the Bradford method with bovine serum albumin as the protein standard (Bradford, 1976).

### Fluorescence measurements

Specific fluorescence was defined as relative fluorescence divided by the corresponding OD_600nm_. Strains carrying the *msfGFP* gene were grown and inducted as described above. One hundred microliter cultures were serially diluted 1:2 with cold M9 medium four times on a microplate on ice (final volume, 300 μL). Fluorescence and cell density (OD_600nm_) measurements were then performed using a Bio TeK Synergy H1 microplate reader (BioTek, Winooski, United States). The reference plasmid-less strain MET 345 was used as a negative control and the corresponding fluorescence intensity was subtracted as background. The excitation and emission wavelengths of msfGFP are 475 and 510 nm. Measurements were performed in triplicate on three biological replicates.

### mRNA concentration and half-life determination

Cells (3 mg dry weight) were harvested and flash frozen in liquid nitrogen, before rifampicin addition (reference point, T0) and 0.3, 0.6, 1, 1.5, 2, 3, 4, 5, 6, 7.5, 10, 12.5, and 15 min after adding rifampicin (500 mg/l) to arrest transcription initiation. Samples were stored at −80°C until mRNA extraction. Stability measurements were performed on six samples (T0 plus five samples extracted after rifampicin addition). mRNA was extracted using the RNeasy Mini extraction kit (Qiagen) according to manufacturer instructions. Briefly, after thawing on ice, samples were centrifuged and the cell pellet was resuspended in 500 μL of RLT buffer supplemented with 0.01% β-mercaptoethanol (Sigma) and the mixture was transferred into a tube containing glass beads (0.1 g). The cells were disrupted with a FastPrep-24 instrument (MP Biomedicals) in three 30 s cycles at 6.5 m/s, with 1 min on ice between each cycle, and centrifuged for 10 min at 13,200 g at 4°C. Any DNA contamination was eliminated using the TURBO DNA-freeTM Kit (Ambion) according to manufacturer instructions. DNase-treated RNA was then quantified using a Nanodrop spectrophotometer (Thermo Fisher Scientific) and the integrity of the RNA was verified using a 2,100 Bioanalyzer (Agilent) with the RNA 6000 Nano LabChip kit (Agilent). The RNA was stored at −80°C until required.

For cDNA synthesis, 5 μg of total RNA was mixed with 1 μL of random primers (500 ng/μL; Life Technologies) and RNase-free water to a final volume of 24 μL. The mixture was heated at 70°C for 5 min and then immediately cooled to 4°C. SuperScript II reverse transcriptase (200 units) was then added with 0.5 M DTT, 15 mM dNTP Mix, and 5x first strand buffer (Life Technologies). Reverse transcription was performed at 42°C for 1 h, followed by inactivation at 70°C for 15 min. The RNA-cDNA hybrids were then degraded by adding 5 U of RNase H (Life Technologies). The cDNA was further purified using Illustra Microspin G-25 columns (GE Healthcare) according to manufacturer instructions.

Quantitative RT-PCR was performed using SYBR Green Supermix (Bio-Rad) on a LightCycler 480 II thermal cycler system (Roche, Mannheim, Germany) with the following temperature program: 5 min at 95°C for pre-incubation; then 45 PCR cycles 10 s at 95°C for denaturation, 10 s at 60°C for annealing, and 10 s at 72°C for elongation. The melting curve analysis protocol consisted of 1 min at 65°C followed by heating to 95°C at a rate of 1°C every 9 s. The qRT-PCR measurements of mRNA stability were performed at the Gentiane platform (INRAE, Clermont Ferrand, France) using the high-throughput Fluidigm method (Biomark). LightCycler quantifications were performed on at least duplicates of each sample combined with at least duplicates of each primer pair. mRNA concentration measurements with the Fluidigm method were performed on triplicates of each cDNA sample combined with 2 to 5 technical replicates of each primer pair. mRNA half-lives were measured in triplicate for each primer pair on one biological replicate.

mRNA concentration measurements by qRT-PCR were performed using the Pfaffl method (Pfaffl, 2001). After normalization to the fold change of the reference gene (*ihfB*, integration host factor β-subunit), results were expressed as the mean n-fold difference (± standard deviation) between the tested strain and the reference strain (MET 346, 5’UTR_33k_30-reporter). The half-lives (t_1/2_) of the mRNA species were obtained from the degradation rates (*k*, *t_1/2_* = *1/k*) after rifampicin addition. The degradation rates were obtained by linear regression of the time evolutions of the cycle threshold (Ct) after rifampicin addition. Since Ct values are very sensitive to small changes in concentration, no attempt was made to estimate delays in transcript degradation after rifampicin addition as this would have been too uncertain. The half-lives were therefore calculated from the linear regression coefficients (the degradation rates, *k*) of the Ct versus time curves provided the fits had *R^2^* > 0.85.

### Primer design

Primers for qPCR were designed using Vector NTI Advance 11 (Life Technologies) with a melting temperature of 59–61°C, a length of 20–22 bp and 50–67% GC content as constraints, leading to amplicons ranging from 75 to 148 bp in size. The reaction efficiency of each pair of primers was tested as a single amplicon on serial dilutions of *lacZ-*, *txAbF-*, or *msfGFP*-containing plasmid, depending on the primer pairs analyzed. Primer pairs were validated if the qPCR efficiency was between 90 and 110% over the dilution range tested. For each reporter gene (*lacZ*, *txAbF*, and *msfGFP*), three primer pairs were designed and distributed equally along the CDSs (beginning, middle, and end of the sequence). The housekeeping gene *ihfB* was used as an internal normalization control.

### Degradational regulation coefficient

The degradational regulation coefficient (ρD) represents the contribution of stability regulation in the control of mRNA concentration (Esquerre et al., 2014). Comparing ρD between two strains with different mRNA concentrations shows if the difference in mRNA concentration is due to a difference in stability. Assuming that a steady state has been reached, differences in ρD between two strains (the strain of interest and the reference strain) can be calculated as the negative value of the slope of the double-logarithmic plot of the degradation rate constant (*k*) against the initial mRNA concentration (before rifampicin treatment) in the two compared strains, ρD = −dlnk/(dln[mRNA]) where dlnk and dln[mRNA] are, respectively, the differences between the logarithmic degradation rate constants and mRNA concentrations between the strain of interest and the reference strain.

## Results

### Effect of 5’UTR sequences on β-galactosidase protein activity

**Figure 1:**
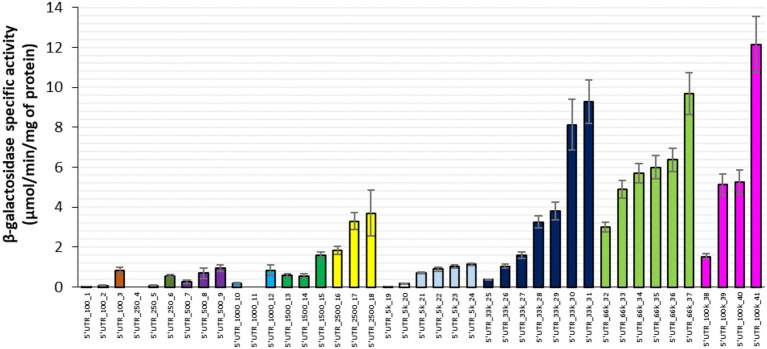
β-galactosidase activity associated with 41 synthetic 5’UTRs ordered by RBS index from 100 to 100,000 AU. Each cluster of columns with the same color corresponds to a specific RBS index series. Error bars represent standard deviations (*n* = 9).

We first designed a set of 41 synthetic 5’UTR sequences, 33 nt long, which in combination with the first 150 nt of *lacZ* cover a wide range of RBS indexes. These were cloned upstream of the *lacZ* reporter gene under transcriptional control of the P_BAD_ promoter and the constructs were transferred into an *E*. *coli* MG1655 derivative strain lacking the chromosomal copy of *lacZ*. We then measured specific β-galactosidase activity (as a good proxy for the β-galactosidase protein level) of the 41 strains after 30 min of induction with arabinose during exponential growth on synthetic glucose medium. Specific β-galactosidase activity varied widely between strains, barely above the detection threshold in some strains, while in other strains, the activity ranged from 0.01 ± 0.00 to 12.14 ± 1.40 μmol/min/mg of protein (Figure 1). The highest and lowest β-galactosidase activities corresponded, respectively, to the highest and lowest RBS indexes (5’UTR_100k_41 and 5’UTR_100_1), but for most of the 5’UTRs, protein activity was not correlated with the theoretical rate of translation initiation. Some 5’UTRs with a high RBS index (e.g., 5’UTR_100k_38) yielded low protein activities, while other 5’UTRs with a low RBS index (such as 5’UTR_2,500_18) yielded high protein activities. Furthermore, several 5’UTRs with the same theoretical translation initiation rate (such as the 5’UTR-33 k series with RBS indexes around 33,000) led to different protein activities (25-fold difference between the lowest and the highest producer of the 33 k series). Altogether, these results show that for most of the 5’UTRs in this experimental model, the theoretical translation initiation rate was not a good predictor of protein activity and by approximation neither of its concentration. This implies that 5’UTR-mediated regulation of translation initiation is not the only determinant of protein level and that additional 5’UTR-associated factors must be involved. We therefore examined the possibility of 5’UTR-associated changes in *lacZ* mRNA concentration.

### Effect of 5’UTR sequences on *lacZ* mRNA levels

**Figure 2:**
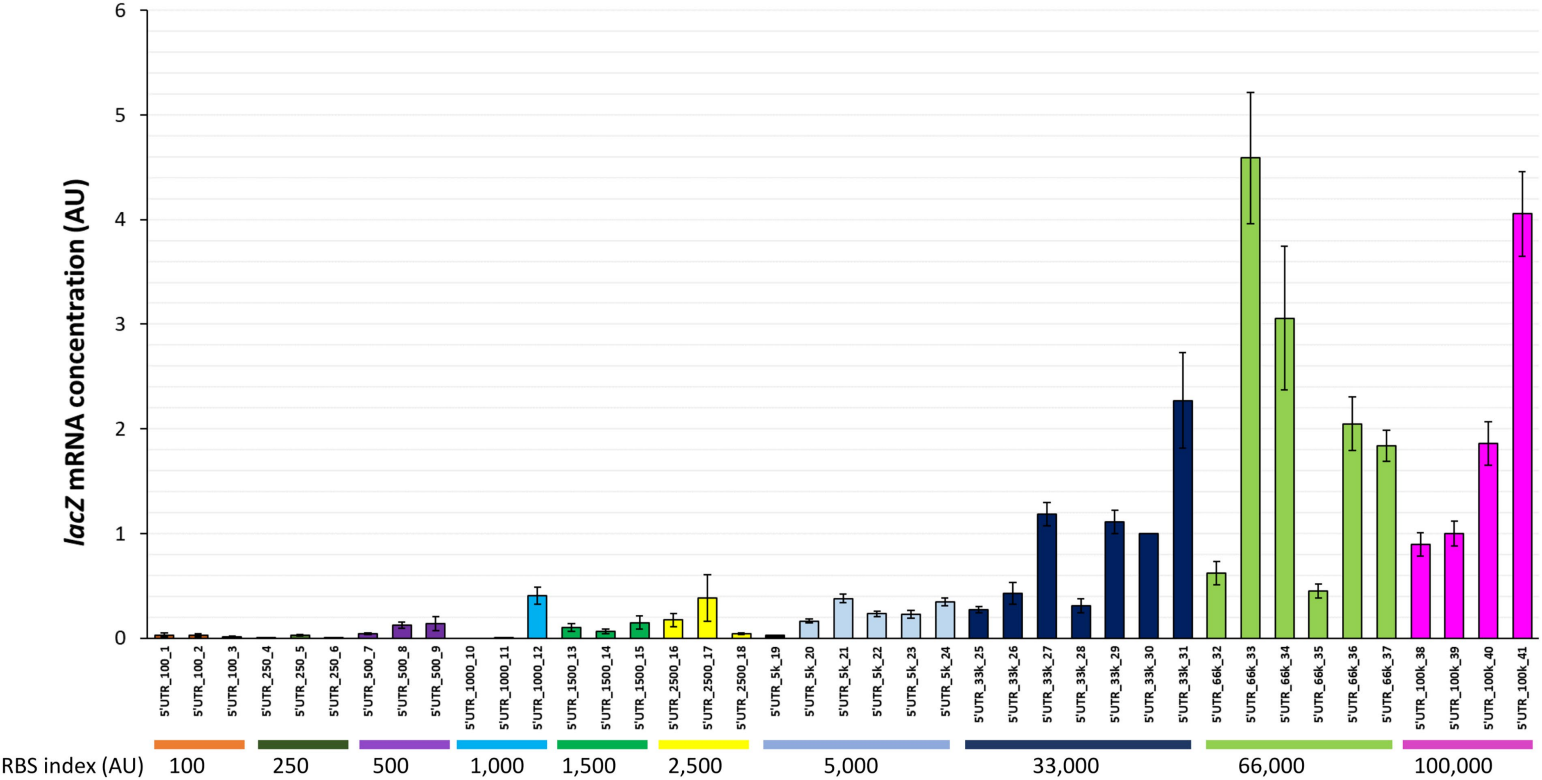
*lacZ* mRNA concentrations associated with 41 synthetic 5’UTRs ordered by RBS index. The *lacZ* mRNA concentration measured in each strain is reported as a fold change relative to the reference strain (5’UTR_33k_30). Error bars represent standard deviations (n = 3).

The *lacZ* mRNA concentration was found to vary considerably between the 41 strains with different synthetic 5’UTRs (Figure 2; ~300-fold difference between the extremes). The mRNA concentrations for some strains (e.g., 5’UTR_250_4 and 5’UTR_1,000_11) were extremely low. Although high mRNA concentrations were only observed for 5’UTRs with high RBS indexes (≥ 33,000), there was no clear correlation between *lacZ* mRNA concentration and RBS index. For instance, strains in the same 5’UTR-33 k series with RBS indexes around 33,000 had mRNA concentrations that differed by a factor of 8 (Figure 2).

**Figure 3:**
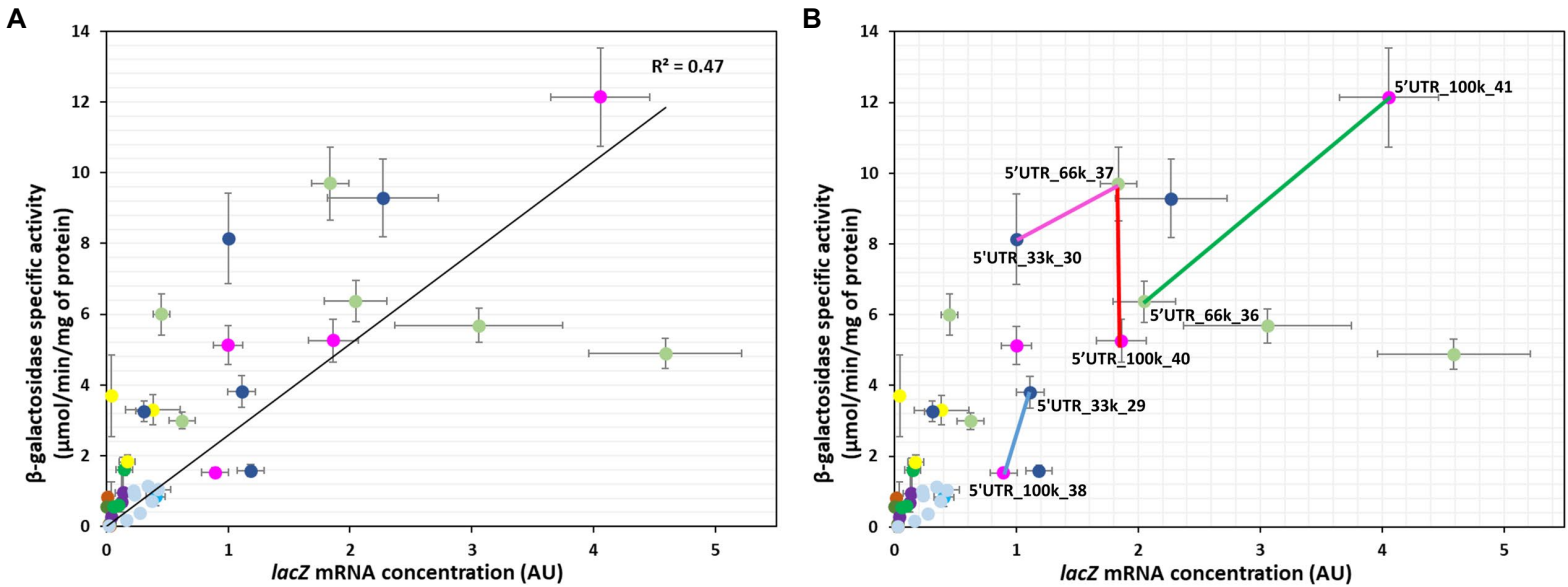
Relationships between β-galactosidase activity and *lacZ* mRNA concentration for the 41 constructs. mRNA concentrations are expressed as fold changes relative to the reference strain (5’UTR_33k_30). **(A,B)** Correlation between protein and mRNA levels (the black line represents the linear regression line, R^2^ = 0.47). The colored lines in part **(B)** highlight examples in which differences in protein levels are mainly due to translation regulation (red), mainly due to mRNA concentration regulation (green), or due to a combination of translation and mRNA concentration regulation acting codirectionally (blue) or in opposite directions (pink). Error bars represent standard deviations (*n* = 3).

We next examined the relationship between protein activity and mRNA level to explore how changes in mRNA concentrations affect the activity of the protein and by approximation its concentration (Figure 3A). As expected (Guimaraes et al., 2014), β-galactosidase activity was positively related to *lacZ* mRNA levels (R^2^ = 0.47; Figure 3A). The moderate correlation suggests that translation and mRNA concentration regulation are both involved in the regulation of protein activity. Pairwise comparisons between the 5’UTRs revealed various scenarios. There were pairs in which the concentration of mRNA was the main determinant of protein activity. For instance, 5’UTR_66k_36 and 5’UTR_100k_41 differed by a factor of 2 in both mRNA level and protein activity (green line, Figure 3B). The relationship for other pairs, indicating no association between mRNA concentrations and protein activities, suggests in contrast that translation is the main determinant of protein activity. This was the case for 5’UTR_66k_37 and 5’UTR_100k_40 for instance (vertical red line, Figure 3B). The data for most pairs of 5’UTRs point toward a combination of translation and mRNA concentration regulation of protein activity, acting in the same or opposite directions. For 5’UTR_33k_29 and 5’UTR_100k_38 (blue line, Figure 3B), the difference in protein activities (2.5-fold) was twice the difference in mRNA concentrations (1.2-fold), indicating that translation and mRNA concentration regulation both increase protein activity. In contrast, between 5’UTR_33k_30 and 5’UTR_66k_37 (pink line, Figure 3B), the increase in the protein activity (+20%) was four times lower than the increase in the mRNA concentration (+80%), indicating that the difference in translation between these strains counteracts the positive effect of the increased mRNA concentration.

Altogether these results demonstrate that 5’UTR sequences affect gene expression both at the translational level and through mRNA concentrations. Intracellular mRNA concentrations are the balance of synthesis *via* transcription and degradation. Since all the constructs had the same level of transcription induction, we next investigated 5’UTR-mediated changes in mRNA stability.

### Effect of 5’UTR sequences on mRNA stability

**Figure 4:**
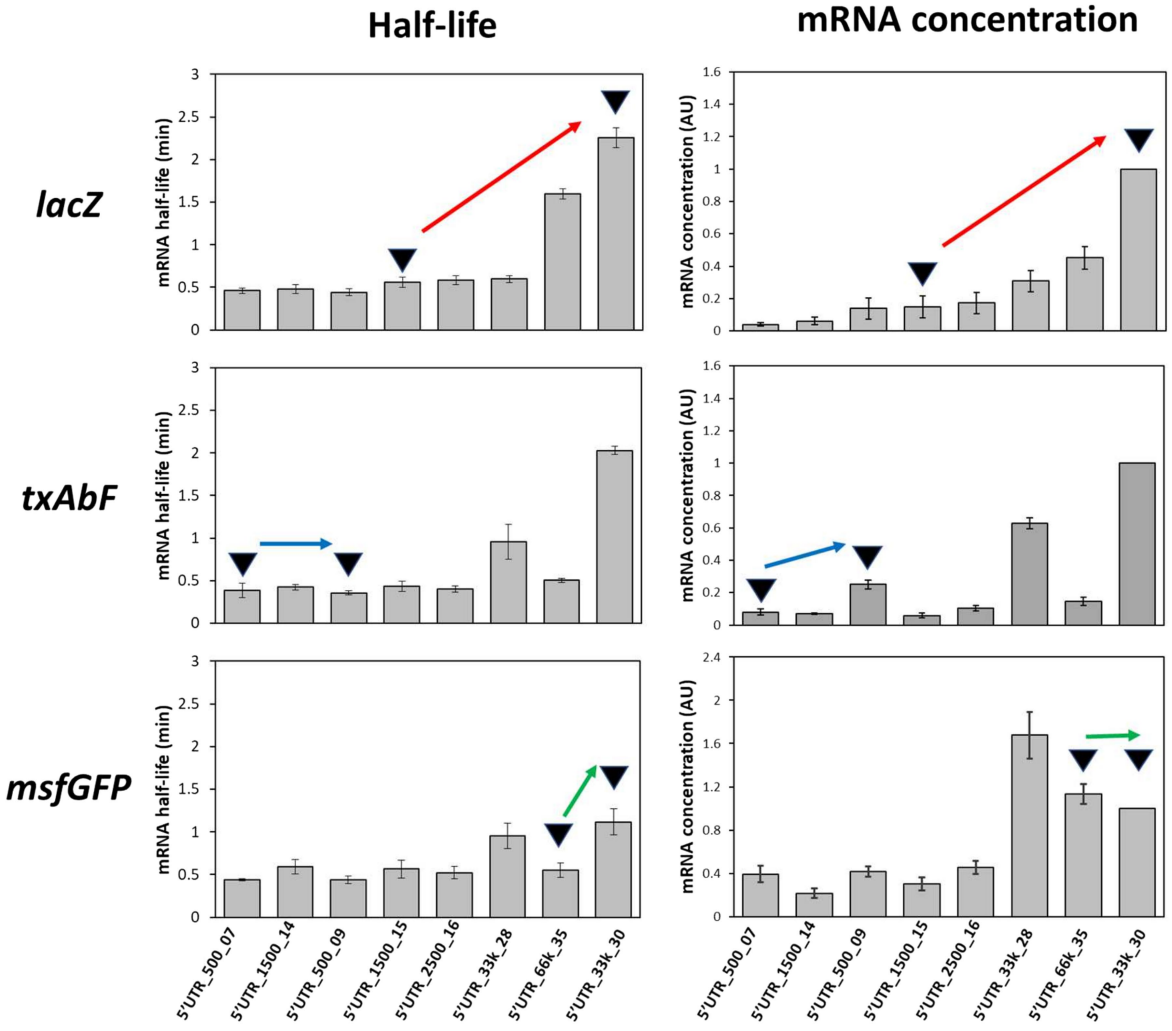
Half-lives of mRNAs fused to the eight selected 5’UTRs and graphical interpretation of the effects of the 5’UTRs on mRNA stability and concentration. Red arrow, an increase in mRNA half-life associated with an increase in mRNA concentration; blue arrow, similar mRNA half-lives but an increase in mRNA concentration; green arrow, a longer mRNA half-life not reflected by an increase in mRNA concentration. Error bars represent standard deviations (*n* = 3).

mRNA stability measurements were performed for 8 of the 41 synthetic 5’UTR sequences with a very high correlation between β-galactosidase expression and *lacZ* mRNA concentrations (R^2^ = 0.91), indicating a predominance of mRNA concentration regulation (5’UTR_500_07, 5’UTR_1,500_14, 5’UTR_500_09, 5’UTR_1,500_15, 5’UTR_2,500_16, 5’UTR_33k_28, 5’UTR_33K_30 and 5’UTR_66k_35; Supplementary Figure S1A). The decrease in *lacZ* mRNA concentration over time was measured after transcription initiation was blocked by adding rifampin. The half-life of *lacZ* mRNA varied between 0.4 ± 0.1 min and 2.3 ± 0.1 min in these constructs (Figure 4), confirming that 5’UTR sequences affect the stability of the corresponding transcript.

We next analyzed whether these changes in mRNA stability depended on the nature of the downstream reporter gene. Half-life measurements were repeated (Figure 4) after replacing the downstream gene (*lacZ*) with either *txAbF* or *msfGFP*. As observed with *lacZ*, the half-lives of *txAbF* and *msfGFP* mRNAs differed between strains with different 5’UTRs. Although 5’UTR_33k_30 was associated with the highest mRNA half-lives with all three reporter genes, the ranking of the 5’UTRs and the magnitude of the variations in half-life (5-fold for *lacZ*, 6-fold for *txAbF*, and 2.5-fold for *msfGFP*) differed between reporter genes.

These results show for three different reporter genes that changing the 5’UTR sequence can affect mRNA stability. However, the magnitude of this effect depends on the nature of the downstream reporter gene.

### Effect of 5’UTR-mediated changes in mRNA stability on mRNA concentration

We then sought to determine whether 5’UTR-dependent changes in mRNA stability could explain differences in mRNA concentrations and protein activity. We first verified for the eight selected 5’UTRs that protein activity/level and mRNA level were as highly correlated with *txAbF* and *msfGFP* as reporter genes as with *lacZ* (Supplementary Figures S1B,C), confirming that protein expression was mainly regulated by the mRNA concentration. It can be noted that both the good protein producers (5’UTR_66k_35 and 5’UTR_33k_30) and the low protein producer (5’UTR_2,500_16) contained the GGAGG SD sequence (Supplementary Table S1) indicating that the presence of a perfectly complementary SD sequence alone does not predict the protein level. We then investigated how changes in mRNA concentration were related to changes in mRNA stability. Graphical comparisons for the eight 5’UTR sequences and the three reporter genes revealed (i) variations in mRNA concentration mirroring variations in mRNA stability (red arrow, Figure 4); (ii) variations in mRNA concentration unrelated to variations in stability (blue arrow, Figure 4), which must therefore be due to differences in transcription; and (iii) similar mRNA concentrations despite variations in mRNA stability (green arrow, Figure 4), indicating that changes in transcription must counteract the effect of increased mRNA stability.

Degradational regulation coefficients (ρD) were calculated to quantify the role of mRNA stability in determining mRNA concentration (Table 1). For a given reporter gene, the contribution of mRNA stability to regulating mRNA concentration was found to vary between 5’UTR sequences. For example, *txAbF* mRNA stability strongly contributed to mRNA concentration regulation with 5’UTR_500_09 and 5’UTR_33k_30, contributed only partially with 5’UTR_500_07, 5’UTR_2,500_16 and 5’UTR_66k_35, and was not involved at all with 5’UTR_1,500_14 and 5’UTR_1,500_15. The role of mRNA stability in regulating mRNA concentration also varied with the reporter gene for a given 5’UTR. For example, changes in mRNA concentration with 5’UTR_500_09 were associated with changes in mRNA stability for *txAbF* (strongly) and *msfGFP* (partially), but not for *lacZ*. Note that none of the 5’UTRs were associated with stability-related control of mRNA concentration for all reporters, while with 5’UTR_1,500_14 and 5’UTR_1,500_15, no regulatory effect of mRNA stability on mRNA concentration was observed for any of the reporters.

**Table 1:** Degradational regulation coefficients (ρD) of constructs with eight different 5’UTRs and three reporter genes.

5’UTR	*lacZ*	*txAbF*	*msfGFP*
5’UTR_500_07	0.13	0.44	0.54
5’UTR_1,500_14	0.13	0.37	0.23
5’UTR_500_09	0.37	1.08	0.56
5’UTR_1,500_15	0.08	0.33	0.31
5’UTR_2,500_16	0.03	0.48	0.46
5’UTR_33k_28	–	–	–
5’UTR_66k_35	2.6	0.44	1.39
5’UTR_33k_30	1.13	1.61	0.31

5’UTR_33k_28 was used as a reference. ρD values are classified in three groups: high (ρD > 0.6, in red), indicating that stability regulation is the primary factor explaining variations in mRNA concentration; low (ρD < 0.4, in blue), indicating that stability regulation does not significantly contribute to variations in mRNA concentration; and intermediate (0.4 < ρD < 0.6, in green), indicating that stability regulation and transcription regulation contribute at similar levels to variations in mRNA concentration.

**Figure 5:**
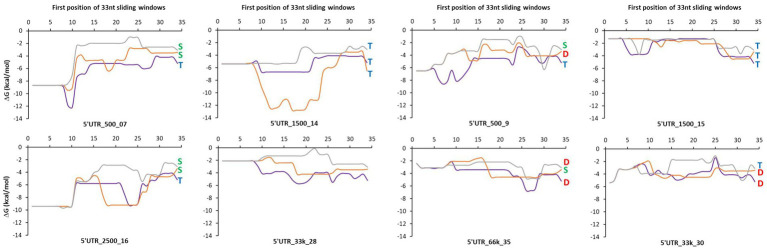
A sliding window analysis of the minimum free energy in the translation initiation region. A 33-nucleotide long sliding window was applied in the translation initiation region (TIR) of each mRNA of reporter genes fused to the eight selected 5’UTRs. The TIR sequence (−33 to +33 bp region relative to the start codon) comprises the synthetic 5’UTR and the first 11 codons of the downstream gene. The minimum free energy was calculated in each window using the mfold software. The minimum free energies of *lacZ* are represented by the purple curve, of *txAbF* by the orange curve and of *msfGFP* by the gray curve. Blue T letter: mainly a transcriptional control; Green S letter: a shared transcriptional and degradational control; Red D letter: mainly a degradational control. 5’UTR_33k_28 was used as a reference for the study of the degradational/transcriptional controls.

To examine whether the type of control of mRNA concentration was related to sequence features, we first analyzed the minimum free energy of a sliding window in the translation initiation region (TIR) that comprises the synthetic 5’UTR and the first 11 codons of the downstream gene (−33 to +33 bp region relative to the start codon; Figure 5). The lower the value of the ΔG, the more the formation of strong secondary structures is possible. Transcriptional control was associated with both the absence and presence of strong secondary structures in the TIR sequences (5’UTR_1,500_14 and 5’UTR_1,500_15, respectively). Degradational control appeared to be present only in the absence of strong secondary structures in the TIR sequences (local ΔG never less than −7 kcal/mol for 5’UTR_500_9 for *txAb*F, 5’UTR_66k_35 for *lacZ* and *msfGFP* and 5’UTR_33k_30 for *lacZ* and *txAbF*). The A/U richness of these TIR sequences was not significantly higher than the others to facilitate cleavage by RNase E, and the complementary SD sequence (GGAGG) and its degenerate forms were both present (Supplementary Table S1).

Altogether, these results show that for 11 of 21 constructs, the 5’UTRs contributed (strongly or partially) to the regulation of mRNA stability, which in turn regulated mRNA concentration. Five of the eight 5’UTR sequences were involved in the regulation of mRNA stability in a downstream gene dependent manner. The absence of strong local secondary structures in their TIR sequences appears to be a necessary but not sufficient element to explain the stability-related control of mRNA concentration.

## Discussion

This study provides evidence that 5’UTR sequences play a key role in regulating gene expression in *E*. *coli*. Our results show that 5’UTRs can influence protein activity/level by regulating translation initiation, mRNA stability and mRNA concentration. We observed that the highest and lowest β-galactosidase protein activities were obtained for 5’UTRs with the highest and lowest theoretical translation initiation rates. Because β-galactosidase protein activity is a good proxy for its concentration, these results confirmed the key role of translation initiation in determining protein levels in these cases. However, for many 5’UTR sequences, the theoretical translation initiation rate was a poor predictor of the resulting protein level. We found that *lacZ* mRNA concentrations are regulated in a 5’UTR-dependent manner, and as shown by the moderate correlation between mRNA and protein levels, that this regulation mechanism in turn influences protein levels, often in association with translational regulation.

For two of the 5’UTRs (5’UTR_66k_35 and 5’UTR_33k_30), changes in *lacZ* mRNA concentrations mirrored strong changes in mRNA stability. The fact that high mRNA concentrations were only observed for 5’UTRs with high RBS indexes supports the hypothesis that translation initiation has an indirect effect on mRNA stability. We also showed that 5’UTR-dependent mRNA stability was dependent on the nature of the downstream gene. Translation initiation region (5’UTR + first 11 codons of the downstream gene) with efficient translation initiation rates recruit more ribosomes to the mRNA, and the high ribosome occupancy may protect the transcript from degradation by RNases, thereby increasing the mRNA concentration (Iost and Dreyfus, 1995). Stability regulation does not appear to be related to the strength of the SD sequence, but it does appear to require the absence of strong local secondary structures in their TIR sequences, an absence that may indeed facilitate translation initiation. A downstream gene-dependent 5’UTR effect on mRNA concentration has been reported previously (Mutalik et al., 2013b), but he did not measure mRNA stability. The context dependence of the 5’UTR effect is a great challenge for synthetic biology because ideally, the effects of standardized regulatory elements should be independent of context. This has motivated the design of new 5’UTR-downstream gene junctions based on overlapping genetic elements with translation coupling, to ensure translation initiation is independent of the nature of downstream genes (Mutalik et al., 2013a).

Interestingly, two 5’UTRs never regulated mRNA concentration through mRNA stability for any of the reporter genes. Although all the studied constructs were controlled by the same promoter and had similar levels of transcriptional induction, the absence of stability regulation indicates that these two 5’UTRs must regulate mRNA concentration through transcription, possibly by acting on RNA polymerase escape from the promoter (Heyduk and Heyduk, 2018). The initially transcribed sequence (ITS) corresponding to the first 20 nucleotides of the 5’UTR has been reported to increase or slow down the escape of the RNA polymerase promoter and thus participate in the initiation of transcription (Heyduk and Heyduk, 2018). Exploring this hypothesis would require measurements of sequence-directed RNA polymerase pausing and RNA/DNA duplex stability in the ITS, which are beyond the scope of this study.

In conclusion, this study demonstrates that synthetic 5’UTR sequences can be used as “regulatory-hubs” of gene expression, as they act at the level of translation and of mRNA degradation and transcription. The complexity arising from overlapping regulation mechanisms and the dependence of the 5’UTR effects on the downstream gene make protein levels difficult to predict for any given construct in the context of synthetic biology. Study of more combinations of 5’UTRs and reporter genes is required to fully understand the rules governing protein level regulation by 5’UTR sequences in *E*. *coli*.

## Data availability statement

The original contributions presented in the study are included in the article/Supplementary material, further inquiries can be directed to the corresponding authors.

## Author contributions

FC performed the experiments and wrote the first draft of the manuscript. SN, LG, and MC-B contributed to conception and design of the study and wrote the manuscript. All authors contributed to manuscript revision, read and approved the submitted version.

## Funding

This work was supported by the French National Research Agency [ANR-18-CE43-0010] and Award of a Doctoral Fellowship (FC) from the China Scholarship Council.

## Conflict of interest

The authors declare that the research was conducted in the absence of any commercial or financial relationships that could be construed as a potential conflict of interest.

## Publisher’s note

All claims expressed in this article are solely those of the authors and do not necessarily represent those of their affiliated organizations, or those of the publisher, the editors and the reviewers. Any product that may be evaluated in this article, or claim that may be made by its manufacturer, is not guaranteed or endorsed by the publisher.

## Supplementary material

The Supplementary material for this article can be found online at: https://www.frontiersin.org/articles/10.3389/fmicb.2022.1088941/full#supplementary-material

SUPPLEMENTARY FIGURE S1 Correlations between protein and mRNA levels of reporter genes fused to the eight selected 5’UTRs **(A)** for *lacZ*, R^2^ = 0.91**; (B)** for *txAbF*, R^2^ = 0.93; and **(C)** for *msfGFP*, R^2^ = 0.87 The eight selected constructs were 5’UTR_500_07, 5’UTR_1,500_14, 5’UTR_500_09, 5’UTR_1,500_15, 5’UTR_2,500_16, 5’UTR_33k_28, 5’UTR_33K_30 and 5’UTR_66k_35. The black lines are linear regression fits. Error bars represent standard deviations (*n* = 9 biological triplicates and technical triplicates for measurements of β-galactosidase and α-L-arabinofuranosidase activity and msfGFP fluorescence/OD, and *n* = 3 for measurements of mRNA concentration).

## References

[ref1] Ah-Seng Y. Rech J. Lane D. Bouet J. Y. (2013). Defining the role of ATP hydrolysis in mitotic segregation of bacterial plasmids. PLoS Genet. 9:e1003956. doi: 10.1371/journal.pgen.1003956, PMID: 24367270 PMC3868542

[ref2] Bissaro B. Saurel O. Arab-Jaziri F. Saulnier L. Milon A. Tenkanen M.. (2014). Mutation of a pH-modulating residue in a GH51 alpha-l-arabinofuranosidase leads to a severe reduction of the secondary hydrolysis of transfuranosylation products. Biochim. Biophys. Acta 1840, 626–636. doi: 10.1016/j.bbagen.2013.10.013, PMID: 24140392

[ref3] Bradford M. M. (1976). A rapid and sensitive method for the quantitation of microgram quantities of protein utilizing the principle of protein-dye binding. Anal. Biochem. 72, 248–254. doi: 10.1016/0003-2697(76)90527-3, PMID: 942051

[ref4] Chowdhury S. Maris C. Allain F. H. Narberhaus F. (2006). Molecular basis for temperature sensing by an RNA thermometer. EMBO J. 25, 2487–2497. doi: 10.1038/sj.emboj.7601128, PMID: 16710302 PMC1478195

[ref5] Dreyfus M. (2009). Killer and protective ribosomes. Prog. Mol. Biol. Transl. Sci. 85, 423–466. doi: 10.1016/S0079-6603(08)00811-8 19215779

[ref6] Esquerre T. Laguerre S. Turlan C. Carpousis A. J. Girbal L. Cocaign-Bousquet M. (2014). Dual role of transcription and transcript stability in the regulation of gene expression in *Escherichia coli* cells cultured on glucose at different growth rates. Nucleic Acids Res. 42, 2460–2472. doi: 10.1093/nar/gkt1150, PMID: 24243845 PMC3936743

[ref7] Folichon M. Arluison V. Pellegrini O. Huntzinger E. Regnier P. Hajnsdorf E. (2003). The poly(a) binding protein Hfq protects RNA from RNase E and exoribonucleolytic degradation. Nucleic Acids Res. 31, 7302–7310. doi: 10.1093/nar/gkg915, PMID: 14654705 PMC291859

[ref8] Gottesman S. Storz G. (2011). Bacterial small RNA regulators: versatile roles and rapidly evolving variations. Cold Spring Harb. Perspect. Biol. 3:798. doi: 10.1101/cshperspect.a003798 PMC3225950 20980440

[ref9] Guimaraes J. C. Rocha M. Arkin A. P. (2014). Transcript level and sequence determinants of protein abundance and noise in *Escherichia coli*. Nucleic Acids Res. 42, 4791–4799. doi: 10.1093/nar/gku126, PMID: 24510099 PMC4005695

[ref10] Hartz D. Mcpheeters D. S. Gold L. (1991). Influence of mRNA determinants on translation initiation in *Escherichia coli*. J. Mol. Biol. 218, 83–97. doi: 10.1016/0022-2836(91)90875-7 1705985

[ref11] Heyduk E. Heyduk T. (2018). DNA template sequence control of bacterial RNA polymerase escape from the promoter. Nucleic Acids Res. 46, 4469–4486. doi: 10.1093/nar/gky172, PMID: 29546317 PMC5961368

[ref12] Holmqvist E. Li L. Bischler T. Barquist L. Vogel J. (2018). Global maps of ProQ binding in vivo reveal target recognition via RNA structure and stability control at mRNA 3′ ends. Mol. Cell 70:e976, 971–982.e6. doi: 10.1016/j.molcel.2018.04.017 29804828

[ref13] Holmqvist E. Vogel J. (2018). RNA-binding proteins in bacteria. Nat. Rev. Microbiol. 16, 601–615. doi: 10.1038/s41579-018-0049-5 29995832

[ref14] Ikeda Y. Yagi M. Morita T. Aiba H. (2011). Hfq binding at RhlB-recognition region of RNase E is crucial for the rapid degradation of target mRNAs mediated by sRNAs in *Escherichia coli*. Mol. Microbiol. 79, 419–432. doi: 10.1111/j.1365-2958.2010.07454.x, PMID: 21219461

[ref15] Iost I. Dreyfus M. (1995). The stability of *Escherichia coli* lacZ mRNA depends upon the simultaneity of its synthesis and translation. EMBO J. 14, 3252–3261. doi: 10.1002/j.1460-2075.1995.tb07328.x, PMID: 7542588 PMC394387

[ref16] Komarova A. V. Tchufistova L. S. Dreyfus M. Boni I. V. (2005). AU-rich sequences within 5′ untranslated leaders enhance translation and stabilize mRNA in *Escherichia coli*. J. Bacteriol. 187, 1344–1349. doi: 10.1128/JB.187.4.1344-1349.2005, PMID: 15687198 PMC545611

[ref17] Komarova A. V. Tchufistova L. S. Supina E. V. Boni I. V. (2002). Protein S1 counteracts the inhibitory effect of the extended Shine-Dalgarno sequence on translation. RNA 8, 1137–1147. doi: 10.1017/S1355838202029990, PMID: 12358433 PMC1370328

[ref18] Kozak M. (2005). Regulation of translation via mRNA structure in prokaryotes and eukaryotes. Gene 361, 13–37. doi: 10.1016/j.gene.2005.06.037, PMID: 16213112

[ref19] Lalaouna D. Simoneau-Roy M. Lafontaine D. Masse E. (2013). Regulatory RNAs and target mRNA decay in prokaryotes. Biochim. Biophys. Acta 1829, 742–747. doi: 10.1016/j.bbagrm.2013.02.013, PMID: 23500183

[ref20] Miller J. (1972). Experiments in molecular genetics Cold Spring Harbor, N.Y.: Cold Spring Harbor Laboratory.

[ref21] Mutalik V. K. Guimaraes J. C. Cambray G. Lam C. Christoffersen M. J. Mai Q. A.. (2013a). Precise and reliable gene expression via standard transcription and translation initiation elements. Nat. Methods 10, 354–360. doi: 10.1038/nmeth.2404, PMID: 23474465

[ref22] Mutalik V. K. Guimaraes J. C. Cambray G. Mai Q. A. Christoffersen M. J. Martin L.. (2013b). Quantitative estimation of activity and quality for collections of functional genetic elements. Nat. Methods 10, 347–353. doi: 10.1038/nmeth.2403, PMID: 23474467

[ref23] Nouaille S. Mondeil S. Finoux A. L. Moulis C. Girbal L. Cocaign-Bousquet M. (2017). The stability of an mRNA is influenced by its concentration: a potential physical mechanism to regulate gene expression. Nucleic Acids Res. 45, 11711–11724. doi: 10.1093/nar/gkx781, PMID: 28977619 PMC5714132

[ref24] Osterman I. A. Evfratov S. A. Sergiev P. V. Dontsova O. A. (2013). Comparison of mRNA features affecting translation initiation and reinitiation. Nucleic Acids Res. 41, 474–486. doi: 10.1093/nar/gks989, PMID: 23093605 PMC3592434

[ref25] Pfaffl M. W. (2001). A new mathematical model for relative quantification in real-time RT-PCR. Nucleic Acids Res. 29:e45, 45e–445e. doi: 10.1093/nar/29.9.e45, PMID: 11328886 PMC55695

[ref26] Prabhakaran R. Chithambaram S. Xia X. (2015). *Escherichia coli* and staphylococcus phages: effect of translation initiation efficiency on differential codon adaptation mediated by virulent and temperate lifestyles. J. Gen. Virol. 96, 1169–1179. doi: 10.1099/vir.0.000050, PMID: 25614589 PMC4631060

[ref27] Prevost K. Desnoyers G. Jacques J. F. Lavoie F. Masse E. (2011). Small RNA-induced mRNA degradation achieved through both translation block and activated cleavage. Genes Dev. 25, 385–396. doi: 10.1101/gad.2001711, PMID: 21289064 PMC3042161

[ref28] Richards J. Belasco J. G. (2019). Obstacles to scanning by RNase E govern bacterial mRNA lifetimes by hindering access to distal cleavage sites. Mol. Cell 74, 284–295.e5. doi: 10.1016/j.molcel.2019.01.044, PMID: 30852060 PMC6541411

[ref29] Romeo T. Babitzke P. (2018). Global regulation by CsrA and its RNA antagonists. Microbiol. Spectr. 6. doi: 10.1128/microbiolspec.RWR-0009-2017, PMID: 29573256 PMC5868435

[ref30] Salis H. M. (2011). The ribosome binding site calculator. Methods Enzymol. 498, 19–42. doi: 10.1016/B978-0-12-385120-8.00002-4 21601672

[ref31] Shine J. Dalgarno L. (1974). The 3′-terminal sequence of *Escherichia coli* 16S ribosomal RNA: complementarity to nonsense triplets and ribosome binding sites. Proc. Natl. Acad. Sci. U. S. A. 71, 1342–1346. doi: 10.1073/pnas.71.4.1342, PMID: 4598299 PMC388224

[ref32] Tucker B. J. Breaker R. R. (2005). Riboswitches as versatile gene control elements. Curr. Opin. Struct. Biol. 15, 342–348. doi: 10.1016/j.sbi.2005.05.003, PMID: 15919195

[ref33] Vakulskas C. A. Potts A. H. Babitzke P. Ahmer B. M. Romeo T. (2015). Regulation of bacterial virulence by Csr (Rsm) systems. Microbiol. Mol. Biol. Rev. 79, 193–224. doi: 10.1128/MMBR.00052-14, PMID: 25833324 PMC4394879

[ref34] Wagner E. G. Romby P. (2015). Small RNAs in bacteria and archaea: who they are, what they do, and how they do it. Adv. Genet. 90, 133–208. doi: 10.1016/bs.adgen.2015.05.001, PMID: 26296935

[ref35] Yarchuk O. Jacques N. Guillerez J. Dreyfus M. (1992). Interdependence of translation, transcription and mRNA degradation in the lacZ gene. J. Mol. Biol. 226, 581–596. doi: 10.1016/0022-2836(92)90617-S, PMID: 1507217

