# Supplementary figures and images for: 5’UTR sequences influence protein levels in Escherichia coli by regulating translation initiation and mRNA stability
Source: Front Microbiol. 2022 Dec 21;13:1088941. doi: 10.3389/fmicb.2022.1088941 (PMC9810816; doi:10.3389/fmicb.2022.1088941)

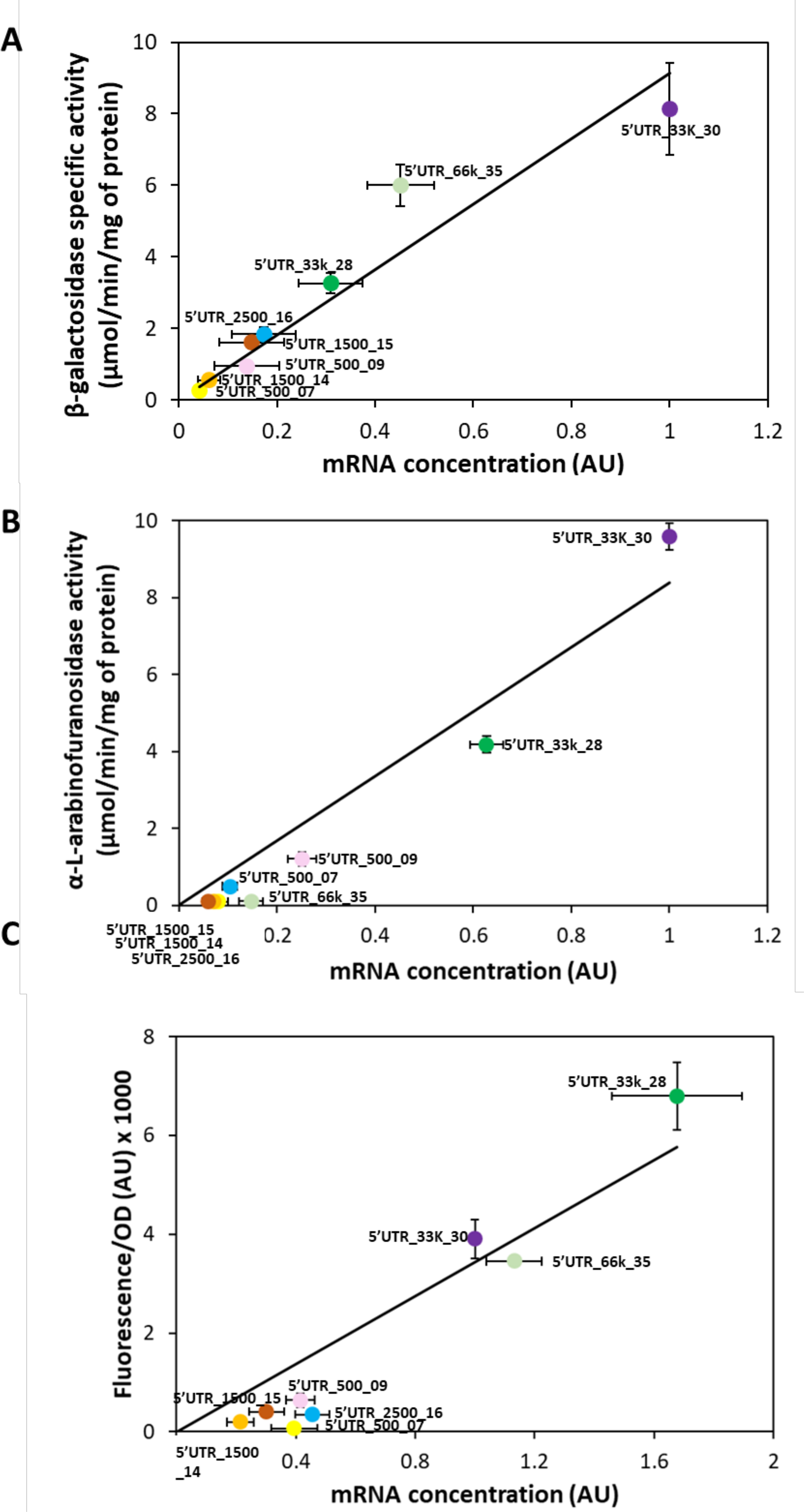

Supplement: SUPPLEMENTARY FIGURE S1 — Correlations between protein and mRNA levels of reporter genes fused to the eight selected 5’UTRs (A) for lacZ, R2 = 0.91; (B) for txAbF, R2 = 0.93; and (C) for msfGFP, R2 = 0.87 The eight selected constructs were 5’UTR_500_07, 5’UTR_1,500_14, 5’UTR_500_09, 5’UTR_1,500_15, 5’UTR_2,500_16, 5’UTR_33k_28, 5’UTR_33K_30 and 5’UTR_66k_35. The black lines are linear regression fits. Error bars represent standard deviations (n = 9 biological triplicates and technical triplicates for measurements of β-galactosidase and α-L-arabinofuranosidase activity and msfGFP fluorescence/OD, and n = 3 for measurements of mRNA concentration). [file Image_1.TIF]
